# Environmental conditions experienced upon first breeding modulate costs of early breeding but not age-specific reproductive output in peregrine falcons

**DOI:** 10.1038/s41598-022-20240-5

**Published:** 2022-09-26

**Authors:** Jabi Zabala, José E. Martínez, Benjamín Gómez-Moliner, Iñigo Zuberogoitia

**Affiliations:** 1 Department of Zoology and Animal Cell Biology, University of the Basque Country, UPV/EHU, C/Paseo de la Universidad 7, 01006 Vitoria‐Gasteiz, Spain; 2 Bonelli´s Eagle Study and Conservation Group, Apdo. 4009, 30080 Murcia, Spain; 3 Estudios Medioambientales Icarus S.L. C/San Vicente, 8. 6 ª Planta. Dpto 8. Edificio Albia I, 48001 Bilbao, Spain

**Keywords:** Ecology, Zoology

## Abstract

Although once considered uncommon, there is growing evidence of widespread senescence in wildlife populations. However, few studies have examined the traits involved, inter-sexual differences, and environmental correlates of age-specific performance in raptors. We studied age-specific reproductive performance and actuarial senescence (decrease in survival probability with age) in a peregrine falcon population monitored for 21 years. We analysed changes with age in the number of offspring produced and incubation start date. We also inspected variation in lifespan and breeding lifespan (number of breeding occasions in a lifetime). In every case, we assessed associations between variations in traits and age, sex, recruitment age, and environmental conditions (cumulative rainfall during breeding season) experienced upon the first breeding attempt. We found scarce evidence for reproductive senescence. Only the incubation start date in females, which was delayed after approximately 8 cy (calendar years), suggested reproductive senescence in our study population. Regarding actuarial senescence, our data did not support it as we only found evidence of higher juvenile mortality. Furthermore, expected lifespan in peregrines recruited at 2 cy was associated with conditions experienced upon the first breeding attempt. The lifespan and breeding career of individuals recruited as yearlings and experiencing low rainfall upon first breeding did not significantly differ from those recruited as adults. However, those recruited as yearlings and experiencing poor environmental conditions upon the first breeding attempt showed reduced lifespan and breeding lifespan.

## Introduction

Contrary to prior beliefs, senescence—the decline in fitness components with age—has been found to be common in wildlife populations^1^. Once accepted as common, the interest shifted from the existence of senescence per se to the prevalence of senescence in different traits and differences in senescence between sexes or individuals and their ecological correlates. Two types of senescence commonly quoted in research are actuarial senescence, the decrease in survival probability with age, and reproductive senescence, a reduction of fertility components at later ages^2–5^.

Experimental evidence has shown that increased reproductive effort can accelerate actuarial senescence^6^. Early life breeding performance can also influence the onset of actuarial senescence^7^ but the effect may only be visible in one of the sexes^3^. For instance, in western gulls (*Larus occidentalis*), males and females that started breeding at early ages showed reduced annual survival^8^, while in other species, only one of the sexes showed earlier senescence associated with breeding in early life^9,10^. Environmental conditions experienced early in life can influence the strength of these trade-offs^11^. In the same line, first breeding occasion can impose selection and there ae possible carryover effects of environmental conditions upon recruitment^12^. Reproductive senescence, in turn, can affect different traits. In birds, senescence associated with early recruitment has been reported in several traits, from clutch size to lifetime reproductive success, but not all assessed traits were always affected^1,4,10^. Thus, while there is a growing wealth of evidence on senescence in wild animal populations, our understanding of it is still fractional, and the results are sometimes contradictory. In the case of raptors, information seems to be particularly scarce. Furthermore, in a recent review of survival estimates in raptors and owls, the only conclusion regarding senescence was that “…*in studies in which adult age-classes were distinguished*, *survival of the very oldest age groups among breeders tended to decline*, *presumably reflecting senescence*…”^13^.

Here, we analysed individual life histories of peregrine falcons (*Falco peregrinus*) from a population monitored over 21 years for age-specific changes in reproductive output and mortality and for evidence of senescence. We investigated survival and reproductive traits, namely lifespan, duration of the breeding career, the number of offspring produced and incubation start date (a proxy to laying date)^14–16^. Furthermore, we accounted for possible variation in age-specific performance related to sex, recruitment age, and conditions experienced upon first breeding attempt.

## Results

### Age-specific reproductive performance analyses

**Figure 1:**
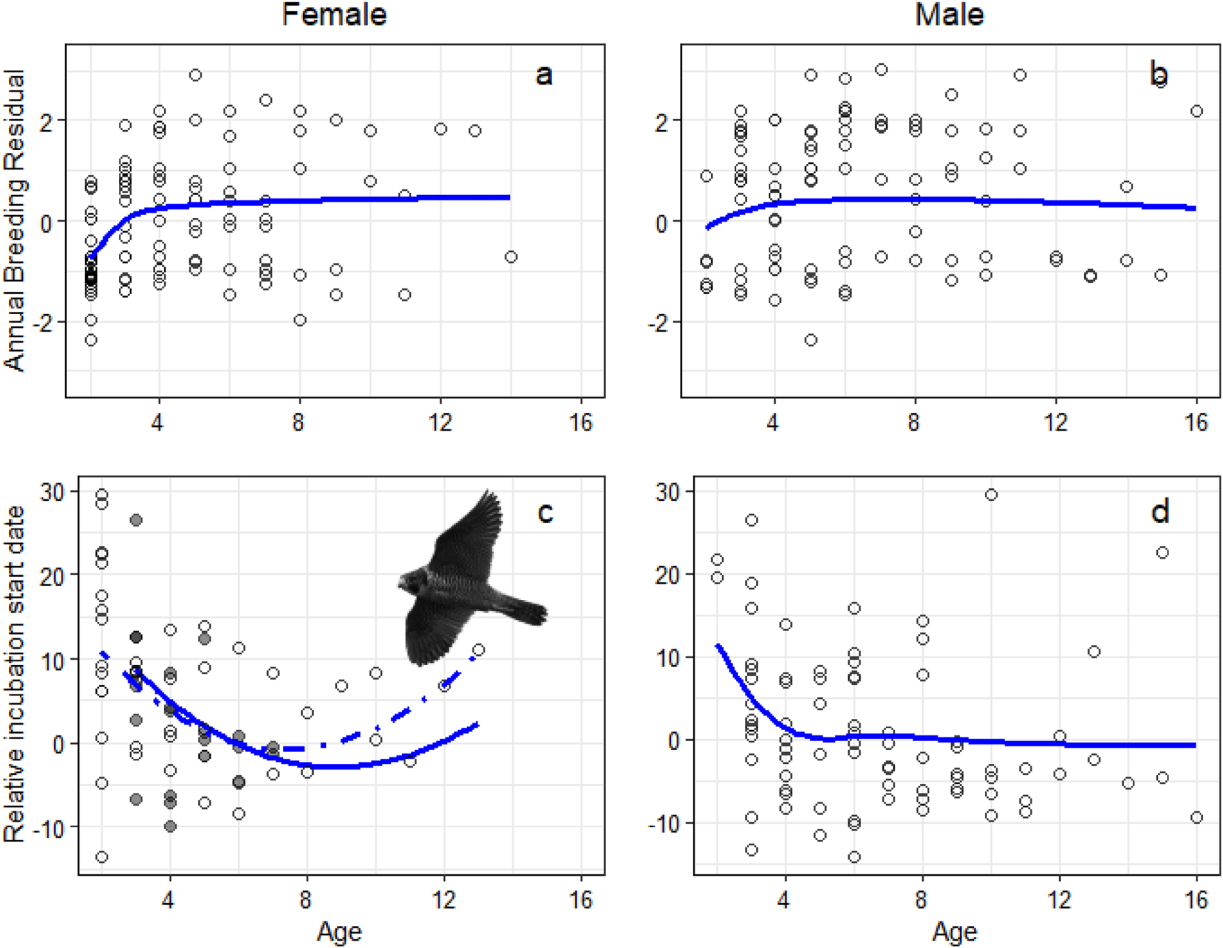
Age specific reproductive performance in peregrines. We show changes in ABR (Averaged Breeding Residual, the number of fledglings produced by a pair minus the average number of fledglings produced by all monitored nests in the population in that year) with for females in (**a**) and for males in (**b**), and variation with age in relative incubation start date for females in (**c**) and form males in (**d**). Points represent observed values, while lines are smoothers fitted over multi-model average predicted values. Ages are in calendar years. The plot for female changes in laying dates, (**d**) includes recruitment age interacting with age; broken lines and empty circles indicate females recruited as yearlings, while the full line and circles show the relationships for females recruited at 3 cy or later. Variation in laying dates show the average trend accounting for conditions experienced upon recruitment. Separate trends for contrasting recruitment conditions are shown in Fig. S1. In some plots, the trends are not centred on zero because ABR and relative laying dates were calculated using all monitored breeding pairs, but age-specific reproductive performance was modelled using only the subset of individuals of known age.

We only found support for reproductive senescence in the incubation start date as females aged. The reproductive output showed increasing values with age in early life but no clear evidence of decline in later life. Multi-model averaged results showed a steep increase in female ABR (Averaged Breeding Residual, defined as the number of fledglings produced by a pair minus the average number of fledglings produced by all monitored nests in the population in that year) in early ages, reaching a plateau at age 4 calendar year (cy, being their first calendar year of a peregrine the year in which it was born; Fig. 1). Males showed an increase in ABR at early ages, although it was less steep than that of females, and their ABR continued to increase, yet very slightly, until approximately 8 cy (Fig. 1). Multi-model averaged evidence indicated that female ABR was substantially improved from ages 2–3 to 4 cy, and in the best model of the set (broken stick at age 3, Table S1), age explained 20.0% of the observed variation in female ABR (*R*^2^_age_ = 0.20). For males, multi-model averaged evidence showed a comparatively less steep increase in reproductive output in early ages and a plateau with no evidence of senesce after that. The best model for male ABR as a function of age was the log model (Table S1) and in it, age accounted for 6.2% of the observed variation in ABR. Relative performance of each of the 12 models assessed for each sex and reproductive parameter are provided in Table S2 and detailed output of each one in Tables S2, S3, S4 and S5.

In contrast, the relative incubation start date suggested senescence in this trait for females (Fig. 1). Juvenile females started incubation comparatively later in the season than prime age females. However, females started incubation at increasing earlier dates as they aged until the age of 7–8 cy and then they started to delay their incubation start date. Regarding the effects of recruitment age, our results suggest that the incubation start dates of females recruited as yearlings showed similar trends in age-specific patterns, but age-related decline seemed stronger for them (Fig. 1). Juvenile males also correlated with later incubation start dates. In their case, the incubation started at increasingly earlier dates until age 4 cy, and after that age, the association of incubation start dates with male age was almost flat (Fig. 1). The best model for the female age and incubation start date included two different slopes with a change point at age 4 cy (min ∆AIC*c* = 2.31, Table S2), and female age accounted for 9.8% of the observed variation (*R*^*2*^_age_ = 0.0–87). For males, the best model considered was broken-stick with two slopes. The change in slope occurred at age 3 cy, when after increasing it became flat (Tables S1 and S3), and age accounted for 13.6% of the observed variation. Peregrines facing rainfall above the average during their first breeding started incubation later regardless of the sex of the inexperienced individual, but the difference with other breeders was relatively small (Fig. S1).

Recruitment age influenced incubation start date in both sexes. The influence of recruitment age was additive for males and interactive with age in females (Sup. Results, Table S6). Thus, all models and plots for age and incubation start date presented here included conditions upon recruitment: interactive with recruitment age for females and as additive covariate for males.

### Factors influencing lifespan and breeding lifespan

**Figure 2:**
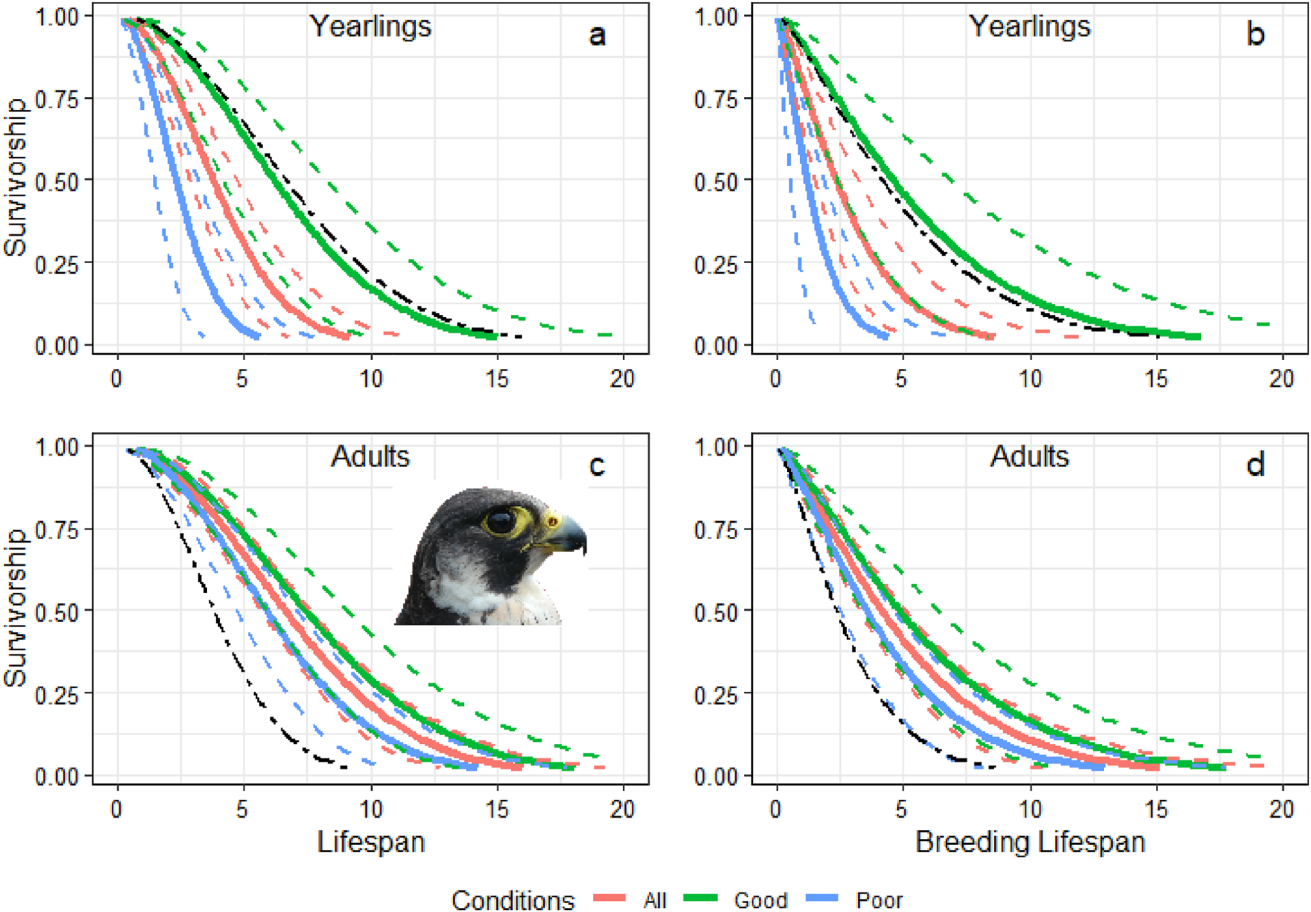
Survivorship and conditions experienced upon recruitment estimated from Accelerated Failure Time (AFT) models. In the top plots, peregrines were recruited as yearlings, and in the bottom ones, those were recruited as adults. The left column shows results for lifespan and the right column results for breeding lifespan. Solid-colour lines indicate survivorship after different conditions upon recruitment (‘All’ model predictions for the average observed conditions upon recruitment; ‘Good’ for average rainfall—standard deviation; ‘Bad’ for average rainfall + standard deviation); broken lines are 95% confidence intervals. The black dot-dashed line shows the all condition average value for the other age-group (peregrines recruited as adults in the plots for these recruited as yearlings and vice versa) and was included to ease comparison. Sample size and their distribution by lifespan lengths are detailed in Table 2. The insert in the bottom-left plot shoes the face of a peregrine falcon, our study species.

Recruitment age and conditions experienced upon recruitment by peregrines recruited as yearlings influenced their lifespan and breeding lifespan. No significant association was observed between recruitment age and age-dependent mortality, but conditions experienced upon recruitment influenced age-related mortality rates and therefore, the average lifespan and breeding lifespan of peregrines recruited as yearlings (Table 1, Fig. 2). The lifespan and breeding careers of individuals recruited as yearlings tended to be shorter compared to those of peregrines recruited as adults, but these differences were not significant. However, lifespan and breeding lifespan of yearling recruits were influenced by weather conditions experienced upon recruitment. Yearlings that experienced poor environmental conditions, i.e., above average rainfall, lived and bred less years, while those recruited as yearlings and experiencing favourable conditions upon the first breeding attempt showed lifespans and breeding lifespans similar to that of peregrines recruited as adults (Fig. 2). This was shown by our best Accelerated Failure Time (AFT) models for lifespan and breeding lifespan retaining in both cases recruitment age and its interaction with conditions upon recruitment (Table 1). The lifespan and breeding lifespan of peregrines recruited as adults, in turn, was not significantly affected by environmental conditions upon the first breeding occasion (Fig. 2).

**Table 1:** Output of best AFT (accelerated failure time) models for peregrine lifespan and breeding lifespan (Brd.-Lifespan).

		Lifespan (*N* = 101, Sh = 0.75)				Brd.-lifespan (*N* = 101, Sh = 0.51)			
		Est	SE	z	*p*	Est	SE	z	*p*
Intercept		2.227	0.371	6	< 0.001	1.693	0.120	14.167	0.001
Intercept	(Yearling)	1.159	0.733	1.58	0.114	− 0.232	0.233	− 0.998	0.318
Rec Cond		− 0.001	0.001	− 1.51	0.132	− 0.079	0.144	− 0.550	0.583
Rec Cond	(Yearling)	− 0.004	0.002	− 2.39	0.017	0.498	0.245	2.034	0.042
Log (Scale)		− 0.289	0.098	− 2.93	0.003	− 0.222	0.096	− 2.315	0.021

The sample size (*N*) and shape parameter (Sh.) of the Weibull function are indicated for each model, as well as the parameter estimate (Est), its standard error (SE), z statistic, and p for each covariate. Rec Cond stands for conditions experienced upon recruitment. With (Yearling), the corrections for the intercept and trends estimated for that group are indicated.

All reported models were based on the Weibull function, which clearly outperformed the Gompertz function (∆AICc > 52 in the most general model), and without territory as random effect as it did not improve the models (∆AICc > 12 in the most general model). The shape parameter of the function was < 1 (Table 1) indicating that hazard decreased with age, and thus lower survival in younger individuals^17^. There was no support for the influence of any of the other covariates assessed on lifespan or breeding lifespan. There was no evidence for differences in lifespan or breeding lifespan between sexes (Table S7). The initial steps of model selection also suggested a correlation between lifespan and breeding lifespan and average ABR. However, the lower productivity of yearlings in their first year^18^ could influence the average ABR and create spurious correlations among them and recruitment age. This could be particularly stark in peregrines recruited as yearlings and with short lifespans. Thus, the analyses were repeated excluding yearling data, and there was no longer found support for a correlation between average ABR and lifespan or breeding lifespan (Table S7).

## Discussion

Our results showed relevant age-specific variation in reproductive output but yielded scarce evidence for senescence, they only suggested reproductive senescence in the female incubation start. In addition, we found evidence of lifespan influenced by recruitment age and conditions upon the first breeding attempt. Individuals recruited as yearlings tended to have shorter lifespans and breeding lifespans, although yearlings experiencing favourable conditions in their first breeding attempt had lifespans and breeding lifespans similar to peregrines recruited at later ages. Yet, we found no evidence for actuarial senescence, as mortality decreased with age.

The improvement in reproductive output, in terms of ABR (Averaged Breeding Residual), early in life is a well-known characteristic of animal populations^19^. However, the pattern of our results after initial increases differed from those reported for other species. In our case, the initial increase phase was short and reached a plateau at the ages of 3–4 cy, with no evidence of senescence after that. In other raptors, the initial increase continued for several years and senescence appeared 2–3 years after the highest reproductive output was achieved, and in some cases, the decline was as steep or more than the initial increase^5,20–24^. The pattern in peregrine males in our study resembled that reported for tawny owl male breeding under strongly variable and cyclic food conditions, with a clear increase in the first years and a slow decrease after that^20^. However, peregrine falcons in our study population were not subject to cyclic prey variation, as in owls foraging on voles. Thus, the plateau after 3–4 years of age could be a trait of the species. A plausible explanation for the lack of evidence of senescence in reproductive output could be the strong territoriality of the species. The number of territories available is limited, and inter-annual variation in the number of territories is small. Thus, there might be strong competition to take over and maintain territories, and adults in their prime could expel pre-senescent individuals from breeding territories. Moreover, during fieldwork, we observed several instances of territory-holders chasing other peregrines out of their territory, which often resulted is serious/fatal injuries^25^. This could not apply to some other raptor species or populations because they are loosely colonial, the number of breeding pairs could be more influenced by food than by the territorial structure of the population, or their densities could be below carrying capacity^21,22,26–29^. However, evidence of reproductive senescence in a numerically stable Eurasian sparrowhawk population and at ages well represented in our study do not agree with that hypothesis^5^. Alternatively, senescence in breeding output in later ages might have been underestimated in our results because later ages are represented in low numbers in our study, as argued for a goshawk population^30^. Furthermore, the few old individuals in our study could represent a selection of higher quality individuals^21^. In any case, the proportion of individuals reaching beyond 12 cy is low, and therefore, the putative effects of senescence breeding output in the population dynamics or ecology would be minimal.

The only other study looking at senescence in laying date (equivalent to incubation start date in our study) on raptors that we are aware of found evidence of a linear increase in laying date with male age but no correlation with female age in a black sparrowhawk population^23^. However, variation in laying date in that population was large, and the resolution for their laying date estimation was low. The authors could only ascertain the month in which the clutch was laid. Several studies on other bird species also reported a “U” shaped relationships of laying date with female age^31–33^. Our results suggest that peregrine females advance their incubation start dates early in their breeding lives as they gain experience up to the ages of 7–9 cy and start gradually delaying the dates, putatively because of senescence. Our results also suggested variation in senescence with recruitment age and environmental conditions upon recruitment. However, the scarcity of data in later life (beyond 6 cy), particularly in females recruited as adults in our sample, prevented further insight. Yearling females showed relatively late incubation start dates, compared to those of other adult females and those of themselves later in life, probably related to delayed gonad maturation^34^. In the case of males, the influence of yearlings (2 cy) and 3 cy old males on incubation start date is probably related to gonadal maturation too. Although the gonads of peregrines can mature at 2 cy, male gonadal maturity takes place later than that of females, and often not until their 3 cy^35^. This explains the low number of males recruited as yearlings and the even lower number of these recruitments producing a clutch (two in our dataset). Males recruited in their third calendar year might still require some time to finish gonadal maturation, resulting in a delay in copulas and clutches. In addition, males support females in the earliest phases of the breeding cycle by providing prey that could help them acquire resources necessary to start laying eggs, and juvenile and young inexperienced males are poor at that^23^. Alternatively, the delay in incubation start date associated with males of 2–3 cy could also be related to their recruitment and decrease/disappearance of the previous male around the early stages of the breeding season. In that case, delayed incubation could be a consequence of the time required to find a new partner and build a bond^25^.

The lifespan and breeding lifespan of peregrines recruited as yearlings (2 cy) was associated with conditions experienced upon recruitment, suggesting that the costs of early recruitment might be modulated by environmental conditions. Previous research reported the influence of natal conditions on lifetime reproductive success and lifespan on owls under cyclic food conditions^4,36^ but not on senescence^20^. We could not test for the effect of natal conditions, as the natal area was unknown for most of the individuals in the study population.

**Figure 3:**
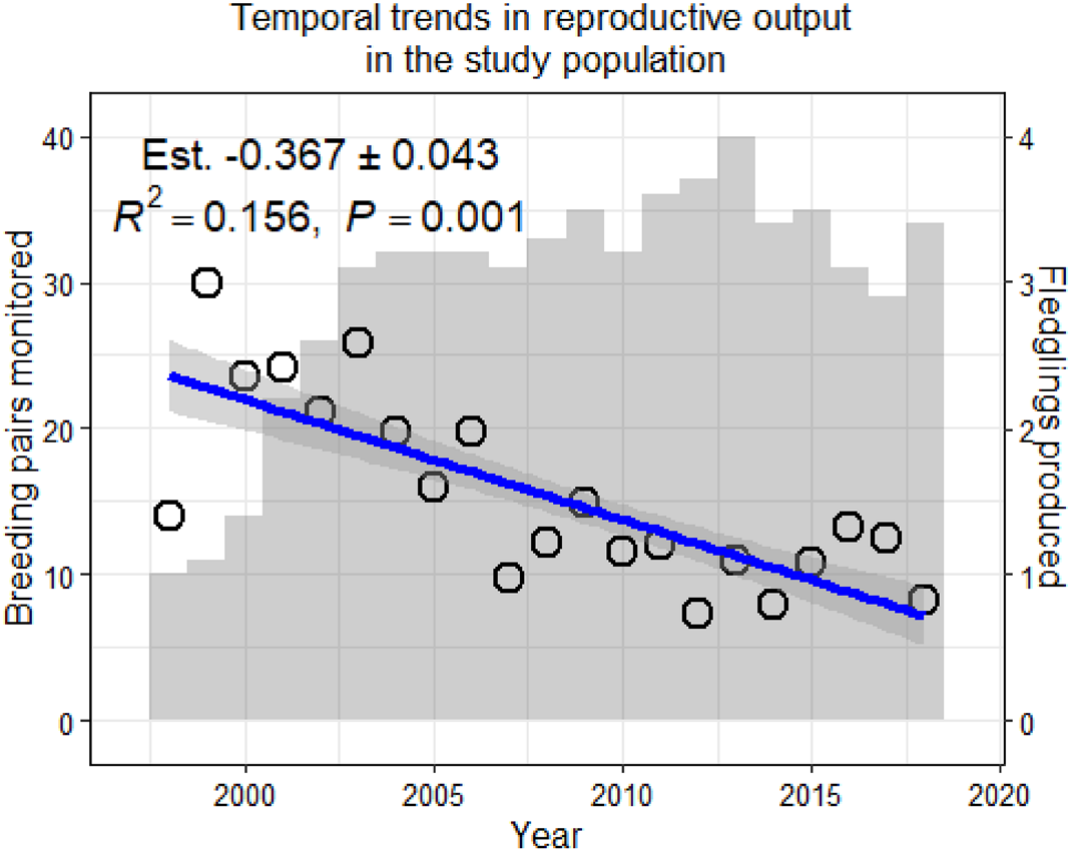
Temporal trends in reproductive output. Values in the plot refer to a linear model of reproductive output as a function of year, and the thick blue line shows the longitudinal trend (with standard deviation shaded) of fledglings produced by breeding individuals (β = −0.367 ± 0.043; *P* < 0.001; N = 617; R^2^ = 0.156; Poisson GLMM with animal Id as random factor). Empty circles show the annual average number of fledglings produced. The shaded histogram in the background shows the annual sample size (breeding pairs monitored).

The interaction between environmental conditions and survival of yearlings appears to contradict previous results from the same population that reported no differences in first year survival of yearling and adult recruits^18^. However, that study only analysed data until 2012, thus including the environmentally more favourable years and excluding the hardest ones (Fig. 3)^25^ in which yearling breeders were more abundant. In any case, these results call for some caution, as they are based on data of only 26 yearlings, of which only seven recruited under favourable environmental conditions (above the average) and four of them lived > 5 years, including two recruited in the first years of the study that lived > 10 years. The other 19 yearlings recruited under harsher conditions, and only three survived > 5 years. These three and a further one were presumably still alive at the end of the study. In any case, those data were censored in our models and thus did not affect our results. Of the other 15 yearlings recruited under worse than average environmental conditions, 10 did not survive their first breeding attempt, and the other five did not survive their second breeding attempt. The latter suggests carryover effects of environmental conditions upon the first breeding attempt in peregrines recruited as yearlings. This early death of yearling recruits that experienced harsh, or average, environmental conditions upon first breeding attempt would also explain the reduction of hazard with age indicated by shape parameters of the Weibull function being less than 1 in both cases^17^ (Table 1). Our results of reduced expected lifespan in animals experiencing harsh conditions upon recruitment fit in the early-late life trade-offs^3^, casting light in the long-term influence of environmental condition experienced upon first breeding attempt for individuals recruiting at early ages.

## Methods

### Study area

We studied the peregrine falcon population of Biscay (Basque Country, northern Spain; 2384 km^2^; 43°12′00″N 3°13′00″W). The territory is hilly and steep, and barely 50 km separate the sea from the highest altitude (1480 m a.s.l.). Forest cultures, pastures, small villages, and densely populated cities shape the landscape. More than 50% of the area is dedicated to forestry, mainly plantations of *Pinus radiata* and *Eucalyptus* spp., at the expense of traditional small-scale farming. The average annual temperature is around 14 °C, and the mean annual precipitation fluctuates between 1200 and more than 2000 mm/m^2^.

### Field procedure

We systematically surveyed he peregrine falcon population annually from 1997 to 2018. In that period, the breeding population fluctuated between 42 and 52 pairs^25^, and our effort focused on 37 territories that were used regularly^37^. Each year, we visited the breeding grounds 30 days before the earliest local laying date recorded for the population (20th February). At this time during the season, breeding pairs can be readily detected since they frequently engage in courtship displays. We observed displays to confirm the presence of territorial breeders, and located crags and eyries used for nesting each year. During these visits, we identified territorial individuals based on alphanumeric coloured rings or high-definition pictures that were inspected for individual phenotypic characteristics and plumage features^38,39^. The age of breeders were determined based on ringing year for those ringed as nestlings. In other cases, the age of individuals breeding for the first time was determined by their typical plumage patterns at 2 (yearlings) and 3 cy^40^. When age could not be determined by these methods, they were considered adults (3 cy or older, 3 cy +). However, we did not included data from these latter individuals in age-related analyses. In the ensuing weeks after the first visit, we revisited breeding grounds to confirm nesting places and the identity of breeders and to monitor egg-laying and incubation onset. Each territory was visited at least twice around the dates in which females tend to lay eggs, which were generally repeated for each female^25^, but in some cases, territories were visited up to eight times to determine egg laying.

When nestlings were 20–30 days old, we visited eyries and ringed nestlings. We later revisited nests with nestlings to determine the number of fledged chicks. When ringing nestlings, we estimated hatching date from nestling growth^25^, and we estimated the incubation start date by subtracting 32 days from the hatching date. This was performed to determine the incubation start dates that were not established by direct observation. All bird handling and ringing permits were issued by the relevant institution (Council of Biscay) and were performed in accordance with relevant guidelines and regulations.

From 1997 to 2018, we monitored 553 breeding attempts, and identified 231 breeders; of these, we obtained or reconstructed the full breeding histories of 102 individuals (see Supplementary Material for a detailed account of data processing). Data from 1997 were not considered in the analyses because it was the first year of the program and few territories were monitored. In the remaining 21 years, we monitored 29.4 ± 8.3 pairs per year (range: 10–40; Fig. 3). We determined the age of 80 individuals (29 males and 51 females) on 216 occasions, for an average of 2.7 ± 2.7 (average ± SD) occasions per individual. Of these 80 individuals, 47 (40 females) entered into the breeding population as yearlings. Notably, yearlings were overrepresented in the sample with regards to their presence in the population because they could be readily aged after plumage features.

The resultant data pool for age-specific reproductive performance analyses of females consisted of 115 observations of reproductive output for 49 individuals and 69 records of incubation start date for 31 individuals. The data set for male age-specific reproductive performance, consisted of 96 breeding output observations from 29 individuals and 80 records of incubation start date from 25 males. In some cases, brood size or incubation start date could not be determined for some individuals of known age, and the effective sample size in each analysis differed from the 216 occasions. In addition, for the analysis of life expectancy, the data consisted of the reproductive life of 102 (57 females and 45 males) peregrines, 33 of which (19 females and 14 males) required censorship (informing the model that the individual was still alive at the end of the study). Those 102 included 75 peregrines (34 females and 41 males; Table 2) recruited as adults (3 cy +) and 27 recruited as yearlings (23 females and 4 males). The distribution of observations of reproductive parameters among different ages are detailed in Table 3.

**Table 2:** Number of peregrines monitored for different consecutive years, split by those monitored over their full lifespan or not (censored), sex, and age at recruitment.

Years monitored		1	2	3	4	5	6	7	8	9	10	11	13	Total
Censored	Yes	1	4	7	9	2	2	2	2	3		1		33
	No	27	15	5	9	3	4	2		1	1		2	69
Sex	Female	18	11	6	11	2	1	3	1	2	1		1	57
	Male	10	8	6	7	3	5	1	1	2		1	1	45
Recruitment age	Adult	14	14	12	16	3	6	3	2	3		1	1	75
	Yearling	14	5		2	2		1		1	1		1	27
Total		28	19	12	18	5	6	4	2	4	1	1	2	102

Data in the first two rows correspond with those used in actuarial senescence analyses.

**Table 3:** data size used in age-specific reproductive performance analyses for each age group.

	Age (cy)	2	3	4	5	6	7	8	9	10	11	12	13	14	15	16	Total
Breeding output	Female	38	18	15	12	9	8	5	3	2	2	1	1	1			115
	Male	6	15	14	13	12	6	7	6	5	3	2	2	2	2	1	96
Incubation start date	Female	15	13	11	9	7	5	3	1	2	1	1	1				69
	Male	2	14	11	6	12	6	7	6	5	3	2	2	1	2	1	80

Age is in calendar years. Females indicate the number of females in each age group included in analyses and males the same for males. In the “Breeding output” row we show the number or records of each age and sex included in Averaged Breeding Residual (ABR) analyses while in the “Incubation start date” we indicate the number of records used relative incubation start date analyses.

### Age-specific reproduction analysis

To account for temporal trends and interannual variation in reproductive output (Fig. 3), we used annual breeding residuals (ABR, defined as the number of fledglings produced by a pair minus the average number of fledglings produced by all monitored nests in the population in that year) as a measure of reproductive performance. The ABR value is indicative of the relative breeding output of the individual compared to the population average; positive values indicate more offspring produced than average, while negative values indicate offspring production below the average in that year. Similarly, we estimated the relative incubation start date by subtracting the average incubation start date in the year from the date of a pair. To analyse age-specific change in reproductive success, ABR, as a function of age, we modelled separately for each sex, using only those individuals for which age could be ascertained (i.e., excluding individuals recruited at an unknown age). Previous analyses showed that individual outperforms territory as explanatory random variable of breeding success, and that when accounting for individual the effect of territory is negligible in terms of AICc^39^. Therefore, to analyse age-specific reproductive performance, we used generalised linear mixed models (GLMMs) with a Gaussian error type (equivalent to linear mixed models), individual identity as random factors and ABR or relative incubation start date as response variables. We favoured this approach over a GLMM with a Poisson error type and a log ling function due to the problems and uncertainty in results inherent to that approach with small counts like clutch-sizes^41,42^. In the same way, we modelled the relative incubation start date as a function of age separately for each sex. Previous studies reported different age-related reproductive performance shapes for each sex^18,20^, we modelled sexes separately and we did not use a single model to analyse it. We favoured an information theoretic approach, assessing several possible shapes and using the evidence contained in the data in favour of each one to weigh them and produce a multi-model averaged shape^43^. Therefore, for both traits, we considered 12 candidate functions to model breeding parameters as a function of age, and the results were combined in a plot averaging model outputs by the support they had from the data (see below). These 12 functions included: (1) a linear model of ABR or relative incubation start date as a function of age; (2) a log model representative of an asymptotic trend in traits with age; (3) a quadratic model to account for curvilinear responses with increases in early life and decreases in later life (or vice versa); and (4–12) nine broken-stick models, also known as piecewise regressions^44^, accounting for two different trends in lifetime with a change point in which the trend changed. In the nine broken-stick models, ages from 3 to 11 cy were evaluated as possible change points. To account for possible effects of selective phenotype appearance/disappearance^12,45^, we assessed the influence of recruitment age and lifespan, either as an additive or interactive effect with age, on the best age function of the 12 assessed (Table S8). We assessed their additive and interactive effects to test not only for different intercepts but also for possible differences on slopes among phenotypes with age. We tested interaction of recruitment age and lifespan only with age parameters and stepwise to avoid over-parameterization given sample size. If support was found for any selective appearance/disappearance effect related to age of recruitment or lifespan, that was included as a covariate in all 12 models. Subsequently, the models were reanalysed, and the analyses were repeated. We also assessed possible carryover effects of environmental conditions upon recruitment^12^. To characterise environmental conditions, we used cumulative rainfall during the breeding season (February to May both inclusive), averaged over 5 weather stations spread over the study area. We assessed the influence of environmental conditions as a covariate (additive and interactive with age) in the best age model of the 12 assessed. If the influence of conditions at recruitment improved the model, we reanalysed the 12 models including is as covariate. We used Akaike’s information criterion corrected for sample size (AICc^43^) to compare models and assess the influence of covariates. To model the shape of the relationships between age and traits, we combined the outputs from the 12 models based on their Akaike weights (AICc_w_). AICc_w_ can be directly interpreted as the conditional probability for each model in the model set^43^. Thus, the predicted values were averaged for each age of the 12 models weighed by the conditional probability of each. If any model obtained essentially no support from the evidence contained in our data (*Δ*AIC*c* > 10; Burnham & Anderson), its contribution to the multi-model weighted averaged model was virtually zero, as any model beyond 7 AIC*c* units of the best model contributes < 1% to the weighted average^43^. Subsequently, we plotted the multi-model averaged predictions and fitted a smoother over it to inspect the average trend of the trait against individual age.

We ran GLMMs using he R package ‘lme4’^46^,. All plots were produced with the R package ‘ggplot2’^47^.

## Factors influencing lifespan and beeding lifespan and actuarial senescence analyses

To model survival probabilities and identify factors affecting breeding lifespan, we used accelerated failure time (AFT) models^17^. AFT models are parametric models that assume survival to follow a parametric distribution and that some covariates may accelerate the failure (death) of individuals. We used lifespan and breeding lifespan, defined as the number of years an individual was alive and the number of years between first and last (inclusive) known breeding attempts respectively, a response variables in AFT models. Monitored peregrines remained in the same breeding territories they first settled and no case of breeders moving to other territory is known in the study area^39^. Therefore, we considered peregrines missing from territories after any breeding attempt to be dead. In AFT models, we assessed the most frequently used functions, Gompertz and Weibull, to model survivorship in wildlife studies^6^. We also assessed the performance of territory as a random factor. Once we determined the best function and whether territory as random factor improved the model or not, we assessed the influence on lifespan and breeding lifespan of sex, averaged relative reproductive output (the average of ABR recorded over the breeding lifespan), and age at recruitment (Table S8). We assessed the possible influence of ABR on lifespan to include possible trade-offs between reproduction and survival^1^. We used lifetime averaged ABR to account for differences in lifespan among individuals. To account for the possible influence and carryover effects of environmental conditions upon the first breeding attempt we also evaluated the influence of environmental conditions upon recruitment and its interaction with age at recruitment on lifespan and breeding lifespan of peregrines. For age at recruitment, we considered two age groups: ‘yearlings’ (individuals that started their breeding career at 2 cy; *N* = 27) and ‘adults’ (individuals that started their breeding career at 3 cy or older; *N* = 75; Table 1). As for analyses of age-specific reproductive performance, we used average cumulative rainfall averaged was used across the study area in the year they were recruited as a proxy for environmental conditions.

To elucidate associations between covariates and lifespan or breeding lifespan, we ran a global model with all the covariates and stepwise dropped non-significant covariates until no further improvement in model fit in terms of AICc was achieved^48^. We run all parametric AFT models using the R package ‘Survival’^49^.

## Supplementary Information

Supplementary Information 1.

Supplementary Information 2.

## Data Availability

The datasets used and/or analysed during the current study are available in Supplemenraty Information 1.
