# Supplementary material for: Environmental conditions experienced upon first breeding modulate costs of early breeding but not age-specific reproductive output in peregrine falcons
Source: Sci Rep. 2022 Sep 26;12:16005. doi: 10.1038/s41598-022-20240-5 (PMC9512846; doi:10.1038/s41598-022-20240-5)

**Supplementary materials for:**

**Environmental conditions experienced upon first breeding modulate costs of early breeding but not age-specific reproductive output in peregrine falcons.**

Authors: Jabi Zabala^1*^, José E. Martínez^2^, Benjamín Gómez-Moliner^1^ and Iñigo Zuberogoitia^3.^

**Data processing**

For survival analyses, we reconstructed the life-histories of adult breeders. As no case of breeding individuals dispersing to other territories was detected (Zabala & Zuberogoitia 2014), we assumed individuals that disappeared from their breeding grounds and were replaced by new breeders to be dead. Some of them might have survived some years as non-breeders elsewhere, but we considered them dead as breeders. In some cases, breeders used alternative nests (Zuberogoitia *et al.* 2015) and depending on weather conditions or the accessibility and the visibility of particular nests used in some years, we could not identify particular breeders, resulting in gaps in the monitoring history of individuals. When the monitoring coincided with the permanence of the same individual in the territory (*N*=6, out of 366 occasions included in analyses) we assumed the individual remained the territory holder during the monitoring gap, as no case of breeders moving to other territory is known in the study area (Zabala & Zuberogoitia 2014). .However, when gaps coincided with the appearance of a new mate/individual in the territory, we discarded the data, with a few exceptions detailed below. We only reconstructed life-histories of those individuals for which we could ascertain when they started their breeding career. These included: (1) individuals that we detected breeding for first time the next year after the previous breeder disappeared (*N*=76). (2) Individuals for which we had a single year gap in monitoring before we first detected them (*N*=11). In these cases, we assumed they started their breeding career in the same year we detected them. And, (3) individuals irrespective to monitoring history of territories, whose plumage patterns suggested they were in their first breeding occasion (i.e. birds aged 2cy, *N*=8, and birds aged3cy, *N*=7). In the same way, we only included in analyses individuals that were replaced by another breeder the next year to their last detection (*N*=54), or with a gap of a single year, assuming the breeder died in the last year it was detected (*N*=15). For eight peregrines, we could ascertain the last year of their breeding career, and other 25 individuals were still alive at the end of the study. We included these 33 individuals in the analyses but specifying the uncertainty about their fate through censoring (informing the model that the individual was still alive and the end of the study).

From 1997 to 2018, we monitored 553 breeding attempts, identified 231 breeders and fully monitored, from their first to last breeding attempt as defined above, 102 of them. We did not consider data from 1997 in analyses because it was the first year of the program and we monitored few territories. In the other 21 years, we monitored 29.4 ± 8.3 pairs per year (range: 10-40; Fig. 1). We could ascertain the age of 80 individuals (29 males and 51 females) in 216 occasions, 2.7 ± 2.7 (average ± SD) occasions per individual. Of these 80 individuals, 47 (40 females) entered as yearlings into the breeding population. Note that yearlings are overrepresented in that sample with regards to their presence in the population because they can be readily aged after plumage features. On average yearlings composed the 3.6 ± 2.9% of the annual breeding population monitored and their proportion showed a slight, yet significant, temporal increase (proportional binomial GLM with the number of yearlings and the number of adult breeders in the monitored territories each year as response variable; *β*= 0.534 ± 0.188, *p* = 0.005; *N* = 21). For instance, the average proportion of breeders that were yearlings in the 1998-2002 period was 2.9 ± 3.2% while in the 2014-2018 period it was 5.2 ± 2.4. Temporal decline in productivity coincided with the increased proportion of yearlings recruited reported above and yearlings could influence our ABR estimates and results. However, a model with time (year as continuous variable) as predictor of average productivity outperformed proportion of yearlings (*Δ*AIC*c* = 12.55), and when assessed together in the same model, the proportion of breeding yearlings in the year did not significantly associate with average productivity (-0.132 ± 0.104; *P*= 0.223, *N*=20). Individual ABR values (with animal as random factor) did neither associate with proportion of yearlings breeding in the year (-2.23^-3^ ± 4.61^-2^; *P*=0.961). This does not mean that the proportion of yearlings has no effect at all on average annual productivity or ABR, but that in this study their effect is imbibed with other trends and it does not seem strong enough to influence or results.

The resultant data pool for senescence analyses of females consisted on 115 observations of reproductive output of 49 individuals and 69 records of incubation start date of 31 individuals. In the case of males, we had 96 observations of breeding output from 29 individual and 80 records of incubation start date from 25 males. Note that, in some cases, we could not determine brood size or incubation start date for some of the known age individuals and effective sample size in each analysis differs from the above mentioned 216 occasions.

In addition, for the analyses of life expectancy, the data consisted of the reproductive life of 102 (57 females and 45 males) peregrines, 33 of which (19 females and 14 males) required censoring. Those 102 included 75 peregrines (34 females and 41 males; Table 1) recruited as adults (3cy+) and 27 recruited as yearlings (23 females and 4 males).

**Functions assessed in AFT models**

The most frequently used functions to model survivorship in wildlife studies are the Gompertz and the Weibull (Boonekamp *et al.* 2014). The Gompertz function models actuarial senescence as an age-dependent multiplier of baseline mortality rate, while the Weibull considers age-related mortality an intrinsic process independent of baseline mortality (Ricklefs & Scheuerlein 2002). As we had no previous assumption of the relationship between baseline and age-related mortality we assessed both functions and selected the better performing one in terms of AICc (Burnham & Anderson 2002). Using the most general model we also assessed the convenience of including territory as random factor in the models.

**Results appearance/disappearance of phenotypes**

We did not find evidence for selective appearance/disappearance of phenotypes in ABR or effect of male age on incubation start date. However, we found evidence supporting an interaction between recruitment age and age in the model for female incubation start date (Table S1). Therefore, all female models of variation in incubation start date with age reported include that interaction. We found no support for an effect of conditions upon recruitment in ABR for males (min ∆AICc= 5.1; *N*= 81) or females (min ∆AICc= 4.21; *N*= 115). On the other hand, we found support for an additive effect of conditions upon recruitment in variation of incubation start date with age in females (2.28 AICc units lower than Null; *N*= 69), while accounting for the recruitment age interaction too. Thus, models presented include conditions upon recruitment as additive covariate too. For males, we found evidence for an interaction between conditions upon recruitment and variation in incubation start date with age (6.06 AICc units lower than Null; *N*= 66). However, only two males recruited as yearlings were included in our models (both in poor years), and we suspected they could be leveraging the results. When we deleted these two records, the best model supported an additive effect of conditions upon recruitment (1.47 AICc units lower than Null and 0.823 lower than the interactive model; *N*= 64; Table S2).

Table S1: Multi-model evaluation of reproductive senescence. Values indicate ∆AICc with regards to the best model in the set. In the broken stick models the value in parenthesis indicates the breaking point as age in CY.

|  | ABR | | Rel. laying date | |
| --- | --- | --- | --- | --- |
| **Model** | Female | Male | Female | Male |
| Broken stick (3) | **0** | 0.91 | 3.67 | **0** |
| Broken stick (4) | 1.99 | 4.58 | **0** | 2.30 |
| Broken stick (5) | 5.11 | 4.57 | 2.22 | 5.48 |
| Broken stick (6) | 9.47 | 3.94 | 3.46 | 7.09 |
| Broken stick (7) | 12.54 | 3.93 | 9.40 | 7.09 |
| Broken stick (8) | 13.11 | 5.40 | 11.07 | 8.00 |
| Broken stick (9) | 13.41 | 6.54 | 11.84 | 7.92 |
| Broken stick (10) | 12.84 | 7.27 | 12.63 | 8.16 |
| Broken stick (11) | 12.86 | 7.30 | 11.89 | 7.57 |
| Linear | 11.37 | 4.99 | 14.86 | 9.55 |
| Quadratic | 14.29 | 11.45 | 12.05 | 12.46 |
| Log | 2.34 | **0** | 5.35 | 4.64 |

Table S2: Detailed model output for models assessing age-specific changes and senescence in breeding output of female peregrines. We show the results of each of the 12 models assessed and used in multimodel inference (for their relative performance, see Table S1). Model indicates the model detailed. In Broken stick models the number indicates the breaking point assessed, and Age 1 and Age 2 the slope for age before and after the breaking point. We show the estimate value for intercept and slopes (Est.), its standard error (S.E.), the value of the t statistic and the associated P value. We do not show intercept variation associated with random factors (individual) because it was 0 in every case.

|  | Models for female age and ABR N=115 from 49 individuals | | | |  |
| --- | --- | --- | --- | --- | --- |
| **Model** | **Covariate** | **Est.** | **S.E.** | **t val** | **P val** |
| Broken stick 3 | Intercept | 0.26 | 0.18 | 1.46 | 0.148 |
|  | Age 1 | -1.10 | 0.25 | -4.38 | <0.001 |
|  | Age 2 | 0.02 | 0.05 | 0.35 | 0.731 |
| Broken stick 4 | Intercept | 0.46 | 0.19 | 2.43 | 0.017 |
|  | Age 1 | -0.62 | 0.13 | -4.59 | <0.001 |
|  | Age 2 | -0.02 | 0.06 | -0.43 | 0.670 |
| Broken stick 5 | Intercept | 0.57 | 0.20 | 2.83 | 0.006 |
|  | Age 1 | -0.42 | 0.09 | -4.45 | <0.001 |
|  | Age 2 | -0.05 | 0.07 | -0.76 | 0.447 |
| Broken stick 6 | Intercept | 0.60 | 0.22 | 2.72 | 0.008 |
|  | Age 1 | -0.30 | 0.08 | -3.94 | <0.001 |
|  | Age 2 | -0.05 | 0.08 | -0.62 | 0.540 |
| Broken stick 7 | Intercept | 0.60 | 0.24 | 2.52 | 0.013 |
|  | Age 1 | -0.22 | 0.06 | -3.49 | 0.001 |
|  | Age 2 | -0.03 | 0.10 | -0.31 | 0.757 |
| Broken stick 8 | Intercept | 0.78 | 0.28 | 2.73 | 0.007 |
|  | Age 1 | -0.18 | 0.05 | -3.37 | 0.001 |
|  | Age 2 | -0.07 | 0.17 | -0.41 | 0.683 |
| Broken stick 9 | Intercept | 0.78 | 0.28 | 2.73 | 0.007 |
|  | Age 1 | -0.18 | 0.05 | -3.37 | 0.001 |
|  | Age 2 | -0.07 | 0.17 | -0.41 | 0.683 |
| Broken stick 10 | Intercept | 0.91 | 0.31 | 2.95 | 0.004 |
|  | Age 1 | -0.17 | 0.05 | -3.48 | 0.001 |
|  | Age 2 | -0.16 | 0.24 | -0.66 | 0.510 |
| Broken stick 11 | Intercept | 0.98 | 0.33 | 2.94 | 0.004 |
|  | Age 1 | -0.16 | 0.05 | -3.40 | 0.001 |
|  | Age 2 | -0.19 | 0.34 | -0.54 | 0.589 |
| Linear | Intercept | -0.67 | 0.21 | -3.24 | 0.002 |
|  | Age | 0.14 | 0.04 | 3.38 | 0.001 |
| Quadratic | Intercept | -1.47 | 0.37 | -3.95 | <0.001 |
|  | Age | 0.49 | 0.14 | 3.40 | 0.001 |
|  | Age quad | -0.03 | 0.01 | -2.56 | 0.012 |
| Log-linear | Intercept | -1.13 | 0.27 | -4.19 | <0.001 |
|  | log (Age) | 0.80 | 0.19 | 4.25 | <0.001 |

Table S3: Detailed model output for models assessing age-specific changes and senescence in breeding output of male peregrines. We show the results of each of the 12 models assessed and used in multimodel inference (for their relative performance, see Table S1). Model indicates the model detailed. In Broken stick models the number indicates the breaking point assessed, and Age 1 and Age 2 the slope for age before and after the breaking point. We show the estimate value for intercept and slopes (Est.), its standard error (S.E.), the value of the t statistic and the associated P value. We do not show intercept variation associated with random factors (individual) because it was 0 in every case.

|  | Models for male age and ABR N=98 from 29 individuals | | | |  |
| --- | --- | --- | --- | --- | --- |
| **Model** | **Covariate** | **Est.** | **S.E.** | **t val** | **P val** |
| Broken stick 3 | Intercept | 0.66 | 0.21 | 3.12 | 0.002 |
|  | Age 1 | -1.43 | 0.59 | -2.41 | 0.018 |
|  | Age 2 | 0.00 | 0.04 | -0.02 | 0.981 |
| Broken stick 4 | Intercept | 0.76 | 0.22 | 3.38 | 0.001 |
|  | Age 1 | -0.51 | 0.27 | -1.92 | 0.058 |
|  | Age 2 | -0.02 | 0.05 | -0.34 | 0.738 |
| Broken stick 5 | Intercept | 0.89 | 0.23 | 3.80 | <0.001 |
|  | Age 1 | -0.36 | 0.17 | -2.16 | 0.033 |
|  | Age 2 | -0.04 | 0.06 | -0.79 | 0.430 |
| Broken stick 6 | Intercept | 1.02 | 0.25 | 4.14 | <0.001 |
|  | Age 1 | -0.29 | 0.12 | -2.41 | 0.018 |
|  | Age 2 | -0.08 | 0.06 | -1.25 | 0.214 |
| Broken stick 7 | Intercept | 1.12 | 0.27 | 4.17 | <0.001 |
|  | Age 1 | -0.23 | 0.10 | -2.43 | 0.017 |
|  | Age 2 | -0.12 | 0.08 | -1.53 | 0.130 |
| Broken stick 8 | Intercept | 1.08 | 0.28 | 3.84 | <0.001 |
|  | Age 1 | -0.17 | 0.08 | -2.08 | 0.040 |
|  | Age 2 | -0.13 | 0.09 | -1.38 | 0.170 |
| Broken stick 9 | Intercept | 1.03 | 0.30 | 3.48 | 0.001 |
|  | Age 1 | -0.12 | 0.07 | -1.75 | 0.083 |
|  | Age 2 | -0.13 | 0.11 | -1.18 | 0.242 |
| Broken stick 10 | Intercept | 0.99 | 0.32 | 3.13 | 0.002 |
|  | Age 1 | -0.09 | 0.06 | -1.46 | 0.148 |
|  | Age 2 | -0.13 | 0.14 | -0.95 | 0.343 |
| Broken stick 11 | Intercept | 0.98 | 0.34 | 2.89 | 0.005 |
|  | Age 1 | -0.08 | 0.06 | -1.31 | 0.192 |
|  | Age 2 | -0.16 | 0.18 | -0.86 | 0.390 |
| Linear | Intercept | 0.36 | 0.30 | 1.20 | 0.235 |
|  | Age | 0.03 | 0.04 | 0.79 | 0.432 |
| Quadratic | Intercept | -0.55 | 0.60 | -0.92 | 0.361 |
|  | Age | 0.33 | 0.17 | 1.88 | 0.063 |
|  | Age quad | -0.02 | 0.01 | -1.74 | 0.085 |
| Log-linear | Intercept | -0.06 | 0.47 | -0.13 | 0.899 |
|  | log (Age) | 0.37 | 0.27 | 1.39 | 0.168 |

Table S4: Detailed model output for models assessing age-specific changes and senescence in incubation start date of female peregrines. We show the results of each of the 12 models assessed and used in multimodel inference (for their relative performance, see Table S1). Model indicates the model detailed. In Broken stick models the number indicates the breaking point assessed, and Age 1 and Age 2 the slope for age before and after the breaking point. We show the estimate value for intercept and slopes (Est.), its standard error (S.E.), the value of the t statistic and the associated P value. Variation in the intercept associated with individuals is shown under “Individual” with its standard deviation in parenthesis, while “Res” indicates the residual variation in the in intercept with its standard deviation in parenthesis.

|  | Models for male age and incubation start date N=72 from 24 individuals | | | | | |  |
| --- | --- | --- | --- | --- | --- | --- | --- |
| **Model** | **Covariate** | **Est.** | **S.E.** | **t val** | **P val** | **Individual** | **Res** |
| Broken stick 3 | Intercept | 1.28 | 1.85 | 0.69 | 0.495 | 8.7 (2.95) | 77.87 (8.82) |
|  | Rec. Conds | -1.03 | 1.63 | -0.63 | 0.533 |  |  |
|  | Age 1 | 17.86 | 6.97 | 2.56 | 0.013 |  |  |
|  | Age 2 | -0.15 | 0.39 | -0.38 | 0.708 |  |  |
| Broken stick 4 | Intercept | -1.07 | 2.00 | -0.54 | 0.595 | 10.82 (3.29) | 77.1 (8.78) |
|  | Rec. Conds | -1.30 | 1.64 | -0.79 | 0.435 |  |  |
|  | Age 1 | 6.58 | 2.63 | 2.50 | 0.015 |  |  |
|  | Age 2 | 0.25 | 0.44 | 0.56 | 0.582 |  |  |
| Broken stick 5 | Intercept | -1.32 | 2.13 | -0.62 | 0.539 | 10.23 (3.2) | 80.37 (8.97) |
|  | Rec. Conds | -1.26 | 1.68 | -0.75 | 0.458 |  |  |
|  | Age 1 | 3.11 | 1.60 | 1.95 | 0.056 |  |  |
|  | Age 2 | 0.32 | 0.50 | 0.64 | 0.527 |  |  |
| Broken stick 6 | Intercept | -1.21 | 2.15 | -0.56 | 0.578 | 11.72 (3.42) | 80.77 (8.99) |
|  | Rec. Conds | -1.51 | 1.69 | -0.89 | 0.382 |  |  |
|  | Age 1 | 1.79 | 1.08 | 1.66 | 0.102 |  |  |
|  | Age 2 | 0.36 | 0.56 | 0.65 | 0.519 |  |  |
| Broken stick 7 | Intercept | -1.94 | 2.38 | -0.82 | 0.419 | 13.52 (3.68) | 79.54 (8.92) |
|  | Rec. Conds | -1.45 | 1.72 | -0.85 | 0.406 |  |  |
|  | Age 1 | 1.50 | 0.89 | 1.70 | 0.095 |  |  |
|  | Age 2 | 0.59 | 0.65 | 0.91 | 0.367 |  |  |
| Broken stick 8 | Intercept | -1.69 | 2.51 | -0.67 | 0.506 | 12.48 (3.53) | 81.21 (9.01) |
|  | Rec. Conds | -1.52 | 1.72 | -0.88 | 0.387 |  |  |
|  | Age 1 | 1.05 | 0.74 | 1.41 | 0.164 |  |  |
|  | Age 2 | 0.59 | 0.75 | 0.79 | 0.432 |  |  |
| Broken stick 9 | Intercept | -2.01 | 2.70 | -0.74 | 0.461 | 12.18 (3.49) | 81.36 (9.02) |
|  | Rec. Conds | -1.47 | 1.72 | -0.85 | 0.401 |  |  |
|  | Age 1 | 0.90 | 0.65 | 1.39 | 0.171 |  |  |
|  | Age 2 | 0.79 | 0.87 | 0.90 | 0.370 |  |  |
| Broken stick 10 | Intercept | -1.92 | 2.92 | -0.66 | 0.514 | 11.1 (3.33) | 82.56 (9.09) |
|  | Rec. Conds | -1.54 | 1.71 | -0.90 | 0.376 |  |  |
|  | Age 1 | 0.72 | 0.59 | 1.22 | 0.228 |  |  |
|  | Age 2 | 0.87 | 1.06 | 0.83 | 0.412 |  |  |
| Broken stick 11 | Intercept | -2.48 | 3.20 | -0.78 | 0.442 | 11.24 (3.35) | 82.12 (9.06) |
|  | Rec. Conds | -1.54 | 1.71 | -0.90 | 0.375 |  |  |
|  | Age 1 | 0.70 | 0.55 | 1.27 | 0.210 |  |  |
|  | Age 2 | 1.36 | 1.34 | 1.02 | 0.313 |  |  |
| Linear | Intercept | 2.83 | 2.87 | 0.99 | 0.330 | 8.78 (2.96) | 84.37 (9.19) |
|  | Rec. Conds | -1.98 | 1.64 | -1.21 | 0.238 |  |  |
|  | Age | -0.24 | 0.40 | -0.59 | 0.557 |  |  |
| Quadratic | Intercept | 10.50 | 5.62 | 1.87 | 0.066 | 12.46 (3.53) | 80.16 (8.95) |
|  | Rec. Conds | -1.35 | 1.72 | -0.78 | 0.440 |  |  |
|  | Age | -2.60 | 1.53 | -1.70 | 0.093 |  |  |
|  | Age quad | 0.14 | 0.09 | 1.61 | 0.112 |  |  |
| Log-linear | Intercept | 6.94 | 4.82 | 1.44 | 0.156 | 8.59 (2.93) | 83.06 (9.11) |
|  | Rec. Conds | -1.41 | 1.67 | -0.84 | 0.406 |  |  |
|  | log(Age) | -3.28 | 2.70 | -1.21 | 0.232 |  |  |

Table S5: Detailed model output for models assessing age-specific changes and senescence in incubation start date of male peregrines. We show the results of each of the 12 models assessed and used in multimodel inference (for their relative performance, see Table S1). Model indicates the model detailed. In Broken stick models the number indicates the breaking point assessed, and Age 1 and Age 2 the slope for age before and after the breaking point. We show the estimate value for intercept and slopes (Est.), its standard error (S.E.), the value of the t statistic and the associated P value. Variation in the intercept associated with individuals is shown under “Individual” with its standard deviation in parenthesis, while “Res” indicates the residual variation in the in intercept with its standard deviation in parenthesis.

|  | Models for female age and incubation start date N=66 from 30 individuals | | | | | |  |
| --- | --- | --- | --- | --- | --- | --- | --- |
| **Model** | **Covariate** | **Est.** | **S.E.** | **t val** | **P val** | **Individual** | **Res** |
| Broken stick 3 | Intercept | -5.79 | 10.15 | -0.57 | 0.571 | 84.67 (9.2) | 22.03 (4.69) |
|  | Rec. Conds | -0.63 | 1.85 | -0.34 | 0.734 |  |  |
|  | Rec. Age | 4.21 | 4.13 | 1.02 | 0.313 |  |  |
|  | Age 1 | 9.10 | 2.64 | 3.45 | 0.001 |  |  |
|  | Age 2 | 4.06 | 2.33 | 1.75 | 0.089 |  |  |
|  | Rec. Age : Age 2 | -1.95 | 1.10 | -1.77 | 0.084 |  |  |
| Broken stick 4 | Intercept | -1.33 | 10.00 | -0.13 | 0.895 | 79.94 (8.94) | 21.43 (4.63) |
|  | Rec. Conds | -0.62 | 1.80 | -0.35 | 0.731 |  |  |
|  | Rec. Age | 1.35 | 4.01 | 0.34 | 0.738 |  |  |
|  | Age 1 | 1.68 | 6.91 | 0.24 | 0.810 |  |  |
|  | Age 2 | 2.22 | 2.88 | 0.77 | 0.446 |  |  |
|  | Rec. Age : Age 1 | 1.55 | 3.15 | 0.49 | 0.627 |  |  |
|  | Rec. Age : Age 2 | -0.88 | 1.38 | -0.64 | 0.529 |  |  |
| Broken stick 5 | Intercept | -0.15 | 10.24 | -0.02 | 0.988 | 84.33 (9.18) | 21.58 (4.65) |
|  | Rec. Conds | -0.82 | 1.83 | -0.44 | 0.660 |  |  |
|  | Rec. Age | 0.44 | 4.09 | 0.11 | 0.915 |  |  |
|  | Age 1 | 2.61 | 4.25 | 0.61 | 0.543 |  |  |
|  | Age 2 | 1.60 | 4.22 | 0.38 | 0.707 |  |  |
|  | Rec. Age : Age 1 | 0.30 | 1.90 | 0.16 | 0.873 |  |  |
|  | Rec. Age : Age 2 | -0.42 | 2.06 | -0.20 | 0.841 |  |  |
| Broken stick 6 | Intercept | 1.74 | 11.40 | 0.15 | 0.879 | 82.96 (9.11) | 23.03 (4.8) |
|  | Rec. Conds | -0.87 | 1.83 | -0.47 | 0.639 |  |  |
|  | Rec. Age | -0.68 | 4.58 | -0.15 | 0.882 |  |  |
|  | Age 1 | 1.78 | 3.47 | 0.51 | 0.611 |  |  |
|  | Age 2 | -2.17 | 8.57 | -0.25 | 0.802 |  |  |
|  | Rec. Age : Age 1 | 0.31 | 1.54 | 0.20 | 0.841 |  |  |
|  | Rec. Age : Age 2 | 1.61 | 4.25 | 0.38 | 0.706 |  |  |
| Broken stick 7 | Intercept | 2.95 | 12.51 | 0.24 | 0.814 | 81.06 (9) | 25.1 (5.01) |
|  | Rec. Conds | -1.09 | 1.83 | -0.59 | 0.557 |  |  |
|  | Rec. Age | -1.05 | 5.04 | -0.21 | 0.836 |  |  |
|  | Age 1 | 1.76 | 2.92 | 0.60 | 0.550 |  |  |
|  | Age 2 | 1.26 | 0.71 | 1.78 | 0.084 |  |  |
|  | Rec. Age : Age 1 | -0.01 | 1.27 | -0.01 | 0.996 |  |  |
| Broken stick 8 | Intercept | 8.09 | 14.12 | 0.57 | 0.569 | 80.8 (8.99) | 26.42 (5.14) |
|  | Rec. Conds | -1.22 | 1.83 | -0.66 | 0.513 |  |  |
|  | Rec. Age | -3.29 | 5.88 | -0.56 | 0.578 |  |  |
|  | Age 1 | 0.42 | 2.82 | 0.15 | 0.881 |  |  |
|  | Age 2 | 1.49 | 0.94 | 1.59 | 0.122 |  |  |
|  | Rec. Age : Age 1 | 0.43 | 1.26 | 0.34 | 0.732 |  |  |
| Broken stick 9 | Intercept | 12.71 | 15.92 | 0.80 | 0.428 | 81.11 (9.01) | 27.15 (5.21) |
|  | Rec. Conds | -1.30 | 1.84 | -0.71 | 0.486 |  |  |
|  | Rec. Age | -5.36 | 6.83 | -0.79 | 0.435 |  |  |
|  | Age 1 | -0.40 | 2.74 | -0.15 | 0.884 |  |  |
|  | Age 2 | 1.81 | 1.27 | 1.43 | 0.162 |  |  |
|  | Rec. Age : Age 1 | 0.70 | 1.25 | 0.56 | 0.576 |  |  |
| Broken stick 10 | Intercept | 19.09 | 17.71 | 1.08 | 0.286 | 81.37 (9.02) | 28.02 (5.29) |
|  | Rec. Conds | -1.39 | 1.85 | -0.75 | 0.457 |  |  |
|  | Rec. Age | -8.02 | 7.82 | -1.03 | 0.310 |  |  |
|  | Age 1 | -1.25 | 2.67 | -0.47 | 0.643 |  |  |
|  | Age 2 | 1.97 | 1.78 | 1.11 | 0.275 |  |  |
|  | Rec. Age : Age 1 | 0.98 | 1.24 | 0.79 | 0.434 |  |  |
| Broken stick 11 | Intercept | 22.34 | 19.70 | 1.13 | 0.262 | 81.54 (9.03) | 28.08 (5.3) |
|  | Rec. Conds | -1.42 | 1.85 | -0.77 | 0.449 |  |  |
|  | Rec. Age | -9.65 | 8.86 | -1.09 | 0.281 |  |  |
|  | Age 1 | -1.50 | 2.63 | -0.57 | 0.572 |  |  |
|  | Age 2 | 3.36 | 2.93 | 1.15 | 0.260 |  |  |
|  | Rec. Age : Age 1 | 1.06 | 1.23 | 0.86 | 0.393 |  |  |
| Linear | Intercept | 3.22 | 13.98 | 0.23 | 0.819 | 83.55 (9.14) | 28.14 (5.3) |
|  | Rec. Conds | -1.54 | 1.86 | -0.82 | 0.418 |  |  |
|  | Rec. Age | 2.90 | 6.43 | 0.45 | 0.654 |  |  |
|  | Age | 2.38 | 2.54 | 0.94 | 0.354 |  |  |
|  | Rec. Age : Age | -1.35 | 1.21 | -1.12 | 0.271 |  |  |
| Quadratic | Intercept | -18.14 | 35.71 | -0.51 | 0.614 | 82.19 (9.07) | 23.03 (4.8) |
|  | Rec. Conds | -0.90 | 1.82 | -0.49 | 0.626 |  |  |
|  | Rec. Age | 17.76 | 17.38 | 1.02 | 0.313 |  |  |
|  | Age | 10.89 | 14.61 | 0.75 | 0.460 |  |  |
|  | Age quad | -1.21 | 1.45 | -0.83 | 0.410 |  |  |
|  | Rec. Age : Age | -7.58 | 7.17 | -1.06 | 0.297 |  |  |
|  | Rec. Age : Age quad | 0.75 | 0.72 | 1.04 | 0.306 |  |  |
| Log-linear | Intercept | 0.22 | 18.35 | 0.01 | 0.991 | 82.1 (9.06) | 26.21 (5.12) |
|  | Rec. Conds | -1.19 | 1.84 | -0.65 | 0.523 |  |  |
|  | Rec. Age | 5.70 | 8.59 | 0.66 | 0.509 |  |  |
|  | Log (Age) | 6.98 | 11.65 | 0.60 | 0.552 |  |  |
|  | Rec. Age : Log (Age) | -5.28 | 5.45 | -0.97 | 0.338 |  |  |

Table S6: Results of model selection for selective appearance/disappearance of phenotypes and effects of conditions upon recruitment on reproductive senescence. Values indicate ∆AICc with regards to the best model in the set. We assessed Rec. Age (age at recruitment), lifespan and conditions upon recruitment (Rec. Cds) as covariates for Annual breeding residuals (ABR) and relative laying date (R. laying date) as function of age. Base refers to the best age model within the 12 candidate assessed, while Additive and Interactive indicate how we correlated the covariate to age effects in the base model. We only assessed interactive effects with age components and lifespan and recruitment conditions independently of each other to avoid overparameterization. We indicate the sample size (*N*) in each analysis.

|  | **Male** | | | **Female** | |  |
| --- | --- | --- | --- | --- | --- | --- |
| **ABR** | Rec. Age | Lifespan | Rec. Cds | Rec. Age | Lifespan | Rec. Conds |
| *N* | 90 | 69 | 81 | 111 | 61 | 115 |
| Base | 0 | 0 | 0 | 0 | 0 | 0 |
| Additive | 5.17 | 6.26 | 5.10 | 3.28 | 6.44 | 4.21 |
| Interactive | 8.35 | 10.88 | 8.33 | 2.37 | 11.57 | 6.95 |
| **R. Laying Date** |  |  |  |  |  |  |
| *N* | 74 | 53 | 64 | 66 | 37 | 69 |
| Base | 0 | 0 | 0.87 | 6.38 | 0 | 2.29 |
| Additive | 1.05 | 2.81 | 0 | 4.27 | 0.38 | 0 |
| Interactive | 0.5 | 3.87 | 0.54 | 0 | 1.94 | 1.53 |

Table S7: Model selection of factors affecting peregrine survivorship. Values indicate ∆AICc with regards to the best model in the set. We show the results of the models using all individuals and of these not including individuals recruited as yearlings to control for effects of their lower reproductive success. Rec Age stands for recruitment age, Rec Cond for environmental conditions experienced in the first breeding occasions and avg (ABR) and (LRS) for average lifetime ABR and LRS respectively.

| **Model specification** | | |  | **Lifespan** | **Breeding-lifespan** | |
| --- | --- | --- | --- | --- | --- | --- |
|  |  |  | All | No Yearlings | All | No Yearlings |
|  |  |  | 101 | 75 | 101 | 75 |
|  | Null |  | 16.43 |  | 10.09 |  |
| Rec Age * | Rec Cond |  | 4.87 | **0** | 3.56 | **0** |
| Rec Age + | Rec Cond |  | 8.40 |  | 6.27 |  |
| Rec Age * | Rec Cond + | Sex | 6.66 |  | 5.66 |  |
| Rec Age * | Rec Cond + | avg (ABR) | **0** | 1.9 | **0** | 2.09 |

Table S8: covariates assessed for each trait. In ABR and incubation date we did not include sex as covariate because we analysed data independently for each set. AFT includes lifespan and breeding lifespan analyses

| Analysis | Covariates assessed | | | | |
| --- | --- | --- | --- | --- | --- |
| ABR | Age | Recruitment age | Recruitment conditions | Breeding lifespan |  |
| Incubation start date | Age | Recruitment age | Recruitment conditions | Breeding lifespan |  |
| AFT | Sex | Recruitment age | Recruitment conditions | Average lifetime LRS | Average lifetime ABR |

Figure S1. Senescence in laying data under contrasting environmental conditions upon recruitment. Blue lines and points show male data while black/grey ones show female data. Broken lines indicate trends for individuals recruited under relatively poor conditions (rainfall during the breeding season above the average of the study period), and continuous lines indicate senescence of individuals that experienced relatively favourable conditions (rainfall below the average) upon recruitment. Darker points represent observed values of individuals that experienced favourable conditions and clearer points those of individuals that experienced relatively adverse conditions


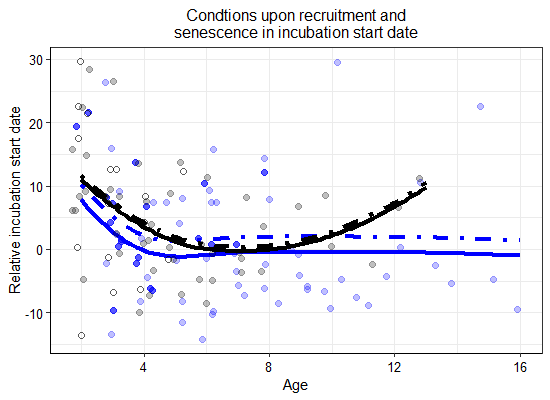

Supplement: Supplementary file 2 — Supplementary Information 2. [file 41598_2022_20240_MOESM2_ESM.docx]
